# The impact of financial deprivation on children’s cybersecurity knowledge & abilities

**DOI:** 10.1007/s10639-022-10908-w

**Published:** 2022-04-18

**Authors:** Suzanne Prior, Karen Renaud

**Affiliations:** 1 Abertay University, Dundee, Scotland; 2 University of Strathclyde, Glasgow, UK; 3 Abertay University, Dundee, UK; 4 Rhodes University, Grahamstown, South Africa; 5 University of South Africa, Pretoria, South Africa

**Keywords:** Passwords, Deprivation, Cybersecurity

## Abstract

Online users require a working knowledge of password “best practice”, as well as the ability to apply such knowledge. Children increasingly operate as independent agents online, and thus also need to be aware of password “best practice”. To meet this need, the Scottish curriculum for excellence includes lessons about password “best practice”. Hence, all Scottish children ought, theoretically, to have similar levels of password-related knowledge. They ought also, by age 8-9, to be able to apply their knowledge. One factor that could deter password-related knowledge acquisition and skill development is financial deprivation. To gauge its impact, we assessed the knowledge and abilities of Scottish 8-9 year old children, in four primary schools, in areas of varying financial deprivation. We uncovered stark differences in knowledge and password retention. There is a clear need for an extra-curricular intervention programme to teach up-to-date password “best practice” and support in developing the required password management skills. This will reduce their online vulnerabilities, whatever their socio-economic background.

## Introduction

The general population needs to be aware of cyber risks and also to understand the measures they can take to resist attacks and prevent breaches (Harknett & Stever, 2009). Given that many attacks occur due to weak passwords (Michael, 2019), everyone needs to know what a strong password looks like, and how to manage all their passwords securely, so that they are able to secure their online accounts. Recently, 500,000 personal Zoom passwords appeared on the dark web (Abrams, 2020), ostensibly to be used by hackers to target personal accounts, evidencing the threats to individual accounts.

If children are operating as semi-autonomous agents online, and managing their own passwords, we have to be sure that they **all** have the required up-to-date knowledge and skills to do so. There is often an assumption that children, as digital natives (Helsper & Eynon, 2010), are well informed when it comes to all kinds of cyber security “best practice”. Yet, Facer and Furlong (2001) argue that the notion of a ‘cyberkid’, who has somehow managed to absorb all necessary knowledge, needs to be reconsidered. According to these researchers, this notion is more anecdotal than grounded in evidence. We thus should not make any assumptions about the knowledge children possess (Bennett, 2012). Recent research confirms that password-related knowledge is not necessarily widespread (Nicholson et al., 2021; Choong et al., 2019).

The Scottish primary school curriculum has been designed to ensure that all children receive the same education in every area. Teachers in Scotland all go through the same training to be accredited to teach. Even so, while children learn at school, they also assimilate knowledge from a variety of other sources. Zevenbergen points out (Zevenbergen, 2007, p. 19) that “*young learners come to early childhood settings with a digital habitus, which is differentially constructed in the home environment and needs to be considered in early childhood practice*.”

**Fig. 1:**
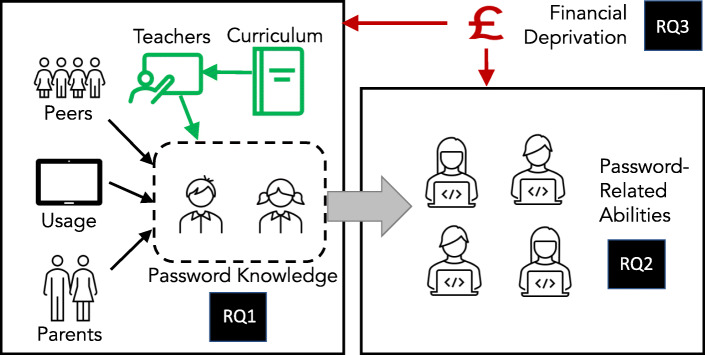
Research Questions 1, 2 and 3

At the moment, we do not know how much young Scottish children know about password “best practice”, We are also not sure how well they are able to apply their knowledge. Finally, we do not know how socio-economic backgrounds impact password knowledge and skills. Hence, the research questions we seek to answer are (Fig. 1):

- What do children know about password “best practice”, and how correct was this knowledge of best practice? (knowledge)
- Can children: (1) create, and (2) recall a “silly sentence” password? (abilities)
- Does financial deprivation impact children’s knowledge and abilities? (impact of financial deprivation)

In Section 2, we discuss the literature on financial deprivation, and its impact on children’s educational attainment. Then, in Section 3, we describe the study we carried out to assess children’s password “best practice” knowledge (RQ1) and skills (RQ2) related to password management. We also show how financial deprivation has an impact on password-related knowledge and abilities (RQ3). Section 4 reports on our findings, and Section 5 discusses our findings, before Section 6 concludes and suggests directions for future research.

## Financial deprivation

Sharma and Atler [2012, p. 546] define financial deprivation as:

“*a psychological state in which people feel financially inferior relative to a salient comparison standard because they perceive a deficit in their financial position.*”

Unemployed people currently make up 4.7% of the UK population.^1^ Financial deprivation undoubtedly has an impact on those who experience it. For example, Waters and Moore (2002) report that financial deprivation has a negative impact on self-esteem, affecting men more than women. Sharma et al. (2014) reported that it also led to a more lenient application of moral standards. Palumbo et al. (2016) found that financial deprivation was a significant predictor of inadequate health literacy. Frasquilho et al. (2016) reported that unemployed adults experienced psychological stress, low life satisfaction and worse mental health outcomes. Vinnerljung et al. (2007) also report on the impact of financial deprivation on both mental and physical health Most concerningly, it led to low levels of well being, and lower educational expectations in the children who experienced deprivation. Indeed, a number of researchers report on the impact of deprivation on educational achievement (Destin et al., 2012; Esposito and Villaseñor, 2019; Dieltiens & Meny-Gibert, 2012).

People with limited financial resources are likely to be more vulnerable, in both the physical (Numans et al., 2021; Saatcioglu & Corus, 2014) and online worlds (Sleeper et al., 2019). To reduce online vulnerability, there are a number of precautions that online users need to take. The UK’s National Cyber Security Centre^2^ offers six actions to be taken to reduce online vulnerability. Three of these are directly related to passwords. It is thus reasonable to assume that poor password practice will increase online vulnerability.

### Knowledge and financial deprivation

Individuals with lower incomes experience greater digital disparities in a variety of areas (Cruz-Jesus et al., 2012). While many of these will be unemployed, this does not apply across the board (Joseph Rowntree Foundation, 2021; Barry, 2020). Many of those living in poverty are employed, but still struggle to make ends meet. The Joseph Rowntree Foundation argue that “*Lack of affordable, flexible childcare and the cost and availability of transport often restrict the hours they can work.*” (Barry, 2020). Many low-paid workers take jobs that fit around their other responsibilities, such as childcare (Hay, 2015). This means that they likely have little spare time, and perhaps will not learn about cyber security precautions, especially when training time in their jobs is often unpaid (Hay, 2015).

With respect to the unemployed, Seabright (2010) explains that they inhabit ‘information islands’ with few bridges to help them access up-to-date information. Those who know a little inform others, but they, too, might possess out-of-date knowledge. This is particularly unhelpful in the cyber security context, a field where best practice and the risk landscape change quickly. It may also be that they are simply exhausted by having to struggle to exist and do not have the bandwidth to worry about online vulnerability. Whatever the reason, it is clear that financial limitations exacerbate vulnerabilities across the board, with cyber being no exception. It is thus possible that financial deprivation will prevent people from gaining access to cyber security related information that they would benefit from if they were employed and participating in training sessions delivered by their employers.

### Children and financial deprivation

Carter (2014) argues that the impact of child poverty on the lives of children and families is “*devastating, long reaching and generational*” (p 3). Walker et al. (2008) also point to the structural and social barriers faced by children in deprived families. Moreover, there is also evidence that family financial difficulties are linked to poorer child mental health outcomes (Kirby et al., 2020).

There is evidence that childhood financial deprivation can impact cognitive development, which leads to children having incomplete or incorrect mental models of essential concepts (Denois et al., 2018). Indeed, Bradshaw [2011 p. 32] argues that “deprived areas act as localised areas of educational disadvantage”. Bradshaw explains that deprived children experience stress and distress as a consequence of their poverty.

Such deprivation could also impact children’s knowledge of password “best practice” and their ability to apply their knowledge. Given the ubiquitous usage of the Scottish Curriculum for Excellence across all Scottish schools, it is reasonable to expect all Scottish children to possess the same level of password-related knowledge by age 9. They ought also to be able to apply that knowledge. However, if financial deprivation impacts these abilities, we would expect to see a difference in the children’s applied knowledge based on the level of deprivation of the school’s geographical catchment area Children from areas of financial deprivation are more likely to be digitally excluded, as compared to children from less deprived areas (Holmes and Burgess, 2020), and it is possible that this could have an impact on their existing cybersecurity knowledge. The (NCSC, 2021) define cybersecurity as:

“*how individuals and organisations reduce the risk of cyber attack.*”

We have adapted this for our target age group:

“*Cybersecurity is how people can reduce the risk of people a victim of cybercrime.*”

Some researchers report on evidence that material conditions have a significant impact on educational attainment (Connell, 1974; Duncan et al., 1994; McKay et al., 1978; Hair et al., 2015; Sosu & Schmidt, 2017; Zhang & Han, 2020). Atkinson and Kintrea (2001) found that merely living in an area of geographically concentrated poverty creates problems for residents, which are likely to affect children too. Ferguson and Michaelsen (2015) confirm this relationship. Marjoribanks (1977) points to the complex interplay between status indicators, family environment and children’s cognitive characteristics. Other researchers highlight a number of other factors that could play a part. For example, Bramley et al. (2007) find that the mere fact of a family owning a home has a positive effect on school attainment. Hanson et al. (2017) report that early adversity plays a role in leading to learning difficulties. Ryan (2016) considers the family religion to play a role here too. A report by the Welsh Government (Hafferty, 2020) suggested that families who did not have access to the internet were more likely to be materially deprived, as compared with those who did have access.

While there is much evidence to suggest that living in financial deprivation has a negative impact on children’s educational attainment and health outcomes, there is also research to suggest that early interventions can effect positive changes. Interventions have been shown to have a positive impact in a variety of different areas including health (Freeman et al., 2016; Kaufman-Shriqui et al., 2016) language (Dobinson and Dockrell, 2021) and problem solving skills (Verma & Verma, 1994). McKay et al. (1978) found that not only did early interventions improve educational outcomes, but that the earlier the intervention, the bigger the impact.

### Password “Best Practice” for children

Choong et al. (2019) found that children’s understanding of password “best practice” was inadequate. In order to delineate the knowledge that different aged children should have, Prior and Renaud (2020) developed an age-appropiate set of ontologies of password knowledge and skills for three different age groups: 4-5, 6-7 and 8-9. However, the ontologies only state what knowledge a child *should* have. Here, we compare actual knowledge with the ‘ideal’ knowledge presented in the ontologies. The ontologies also do not factor in environmental impacts on assimilated (as opposed to taught) knowledge, as depicted in Fig. 1.

## Investigation in primary schools

Children need a working knowledge of password “best practice” principles, as well as the ability to create a strong password and retain it so that they can provide it when required (Prior & Renaud, 2020). All Scottish schools follow the Scottish Curriculum for Excellence, which divides the curriculum into different disciplines. Cyber Security falls within the Technology area. There are five curriculum levels: (1) Early, (2) First, (3) Second, (4) Third and (5) Fourth. Pupils move through the levels at a pace tailored to their particular needs. However, the broad expectation is that they are working at these levels within particular years (see Table 1).

**Table 1:** Curriculum for Excellence Password Benchmarks

Level	Academic Years	Age	Expected Password Knowledge and/or Skills
Early	Nursery - Primary 1	3-6	Logs onto a preferred device with a given password
			Demonstrates an understanding of the importance of passwords and passcodes for example access to the school building
First	Primary 2 - Primary 4	5-9	Demonstrates an understanding for the need for strong password
Second	Primary 5 - Primary 7	8-12	Uses strong passwords
Third	Senior 1 - Senior 3	11-14	None Mentioned

There are benchmarks which pupils are expected to meet within a stage before progressing to the next. Benchmarks relating to passwords are found in the Technology section Education Scotland: Benchmarks technologies ().

Teacher training in Scotland is standardised. All teachers will be trained at a university, as well as completing placements within schools. A teaching degree is either a four year undergraduate study or a one year postgraduate study. In addition, teaching staff within schools will receive relevant training at various periods through the school year.

If we carry out a study to compare children’s knowledge and skills in different schools, and we find differences, we can infer that such differences do not arise from differences in curriculum or teacher training. The differences might come from the teacher’s own understanding of the cyber security arena, their home environment and other sources, such as their peers, TV or online videos. If their educational attainment and cognitive abilities have been impacted by financial deprivation, such children might not have retained the knowledge as well as other children from wealthier homes. The same applies to their ability to apply newly assimilated information.

In the password context, *knowledge* means knowing how to create a strong password, and how to manage it i.e. not sharing it, not writing it down etc. *Abilities* refer to being able to create a password after having received a lesson on how to do this, as well as the ability to retain such a password after a short time lapse.

### Assessing password knowledge & skills

How shall the children’s knowledge be assessed? This can be non-trivial because direct questioning might well change their understanding if we inadvertently frame their responses. We also did not want it to become a testing situation, which they would not enjoy.

Other researchers have experimented with a range of other ways to assess children’s knowledge. Sarti et al. (2018) engaged children using photography which was designed to hear the children’s voices. Their photos revealed critical community issues and triggered critical discussions about the photos.

Kodama et al. (2017), Nicol (2014) and Xu et al. (2009) used drawings to elicit mental models from children. Prokop et al. (2009) explain that the level of students’ existing knowledge around a topic was strongly associated with the details within the drawing they produce when requested to do so. We chose to go with the latter, because of the economic costs of providing children with cameras. Hence, drawings were used to assess the children’s unprompted knowledge of password “best practice”.

To assess their password creation and retention abilities, we wanted them to create a password, and then recall and provide the password again after an interval. This would allow us to test actual ability rather than mere knowledge of what ought to be done in this respect. Children’s memory abilities increase rapidly during their early years before plateauing at the age of 8 and then increasing again during adolescence (Gathercole, 1999). Therefore it should not be an unrealistic expectation for children to be expected to remember a short passphrase after a short delay.

### Recruiting

With the help of a contact at Education Scotland, and our own contacts, we recruited four schools in the North East of Scotland to participate in the study. Over a period of six months, we visited the schools. In some schools we visited two classes so that six classes were involved in the study. Children were aged between 8 and 10 years of age. In total, 141 children participated. The sample was random based on the number of children present in the classes on the day we visited (Table 2).

**Table 2:** Demographics of Schools - adapted from (McLaughlin, 2021)

School ID	School Roll	% Deprivation	State/ Private	% Meeting Literacy Levels
A	100-200	90-100	State	70-80
B	400-500	10-20	State	90-100
C	200-300	70-80	State	80-90
D	100-200	*	Private	*

### Methodology

**Fig. 2:**
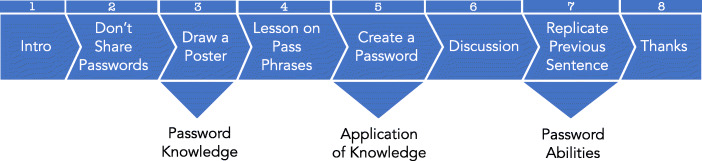
Experiment Stages

The sessions were designed to be a mixture of active learning and listening to the information provided by the researchers. The session was composed of eight stages, as shown in Fig. 2.

#### 1. Introduction:

In each class we were introduced by the class teacher who explained to the children that we were there to teach them about passwords and to see what they already knew about passwords.

#### 2. Admonition:

We were required by our ethics committee to tell children that they should not share their own passwords or anyone else’s during the lesson. We thus commenced by ensuring all children understood this.

#### 3. Draw a poster (assessing knowledge):

The first activity the children completed was to design a poster about passwords in which they would share what they already knew about passwords. They were instructed to make a poster with everything they thought other people should learn about passwords. Children were provided with a piece of A3 paper, happy and sad emoji stickers and colouring pens. This was to replicate similar activities that a child might be used to doing in the classroom.

**Fig. 3:**
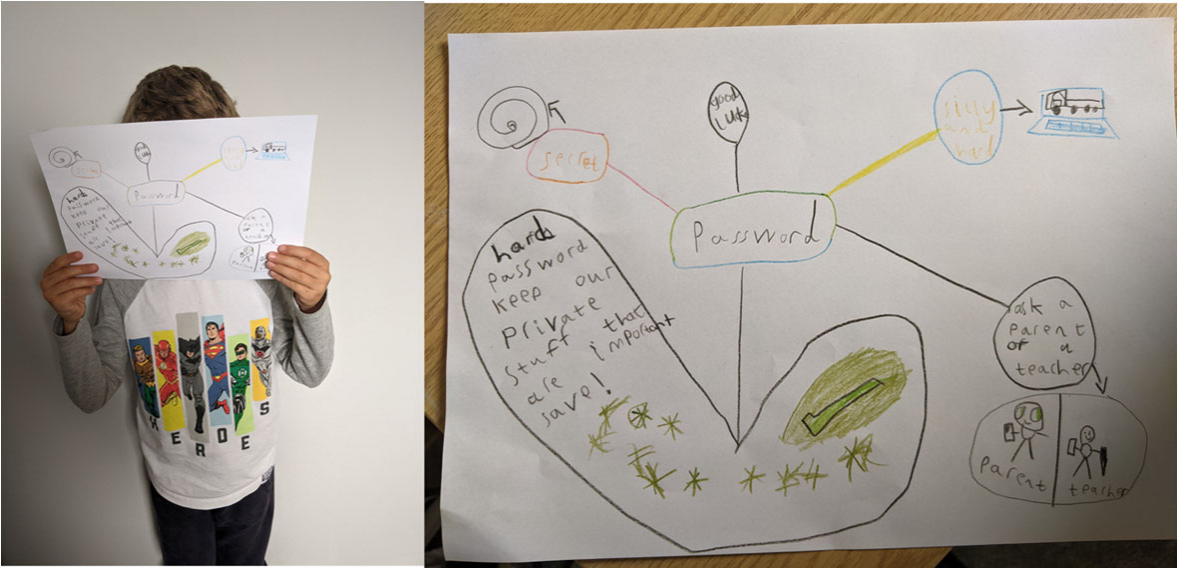
Child with Password Poster (Used with permission from the child’s mother)

By providing new materials, we aimed to increase children’s interest in the activity. This activity lasted 20 minutes. We provided reassurance and clarification where required but neither we nor the teacher provided the children with suggestions about what should appear on the posters (Fig. 3).

#### 4. Introduction to ‘silly sentence’ passwords (new information):

We then explained the principles of a good password and the concept of passphrases, described as a “silly sentence”, was introduced. We were aware that many resources on passwords still referred to the use of complexity to strengthen passwords (Prior and Renaud, 2020) and so we wanted to ensure all children had the same understanding of passphrases. We explained that we were going to now talk about what makes a good password. The children were given the example of “*red fox in the woods*” and it was explained to them why this was a better password than “*Hh234nls3!b31*”. They were taught the principles of a good passphrase: that it should be easy to remember and difficult for other people to guess.

#### 5. Create a ‘silly sentence’ password (ability to create a passphrase):

We asked the children to come up with a silly sentence passphrase. They were asked to write it on a PostIt without letting anyone else see it. They then posted it into a cardboard ‘letter’ box.

#### 6. Discussion:

There was a discussion on password sharing and clarification about whom they could share their passwords with (e.g., with a parent or guardian). The children were asked who they should share their passwords with. This generated a lively discussion. If the children did not engage in the discussion we prompted them with questions such as “do you think you should share with a brother/sister?”.

#### 7. Remember their ‘silly sentence’ password (ability to retain and replicate passphrase):

The children were asked to remember their silly sentence and to write it on a postit note, which was posted into a cardboard ‘letter’ box. (We had given each child two postits with the same number on them, so that we could match their first and second attempts, anonymously).

#### 8. Questions & Thanks:

The children then had an opportunity to ask any questions they had and we ended by thanking them for their participation.

### Ethics

This study was approved by the University of [Redacted]. Both researchers obtained Protection of Vulnerable Groups clearance before the commencement of the studies. A teacher was always present during lessons. All posters and newly-created passwords were anonymous and could not be linked to specific children. Our ethical review board required us to tell children not to share their passwords before the activities commenced.

Because the children retain copyright, we do not include any of the drawings in the paper, except one drawing where the mother explicitly gave permission for us to do so (Stage 2 in Fig. 2).

### Analysis

We now explain how we analysed the posters and the children’s passwords (created and recalled) to answer the research questions.

#### RQ1: Knowledge and correctness thereof

Each poster was checked and any identifying information removed, then given an ID number and digitised. Each piece of information on the poster was entered into a spreadsheet. We then analysed the data as follows.

##### Number of Poster “Best Practice” Principles:

We tallied the number of principles conveyed in the posters (not distinguishing between correct and incorrect).

##### Correctness of Poster “Best Practice” Principles:

In addition to the analysis mentioned above, we counted the number of *correct* principles the children included on their posters. We used Prior and Renaud’s (Prior & Renaud, 2020) ontology to reveal the collective knowledge of each school. The authors coded each poster independently. The inter-rater reliability of the coding was 83.1%.

#### RQ2: Ability to create and retain a password

We copied the two passphrases into a spreadsheet.

##### Password creation:

To test *creation* ability, we worked through the passphrases to see whether it was made up of three or more words without the addition of random characters i.e. tending towards complexity. The total number of participants from each school was used to determine the percentage of correctly created passphrases.

##### Password retention:

To test *retention* ability, we compared their first and second passwords on the postits.

##### Password strength:

The passphrases were entered into the Bennish Calculator (Kennish, 2019) to quantify their strength on a scale of 1 (weak) to 5 (strong). (Those that were not passphrases were not rated).

#### RQ3: Impact of financial deprivation

The results from RQ1 and RQ2 were compared and correlated with the levels of deprivation for each school’s catchment area to reveal differences and similarities in the children’s performance.

## Findings

### RQ1: What did children know about password good practice, and how correct was their knowledge?

This analysis showed that the children did not have an extensive knowledge of password “best practice”. For example, none of the children within this study identified the danger of someone watching while they entered a password, the consequences of their password being leaked or that they should be looking for HTTPS before entering a password. The children also did not know that they should not use the same password everywhere.

Children were most likely to know that a password should be difficult for others to guess - this was often expressed as “*not your name or date of birth*”. However, some posters then suggested ways that this could be obfuscated in predictable ways - for example changing letters for special characters. This would still be considered bad practice. The children from the state school with the least deprivation (School B) were most likely to provide incorrect information, the majority related to outdated complexity requirements for passwords rather than passphrases (Renaud, 2021). Again, not their fault that they had picked up legacy practice, probably from adults in their lives.

**Fig. 4:**
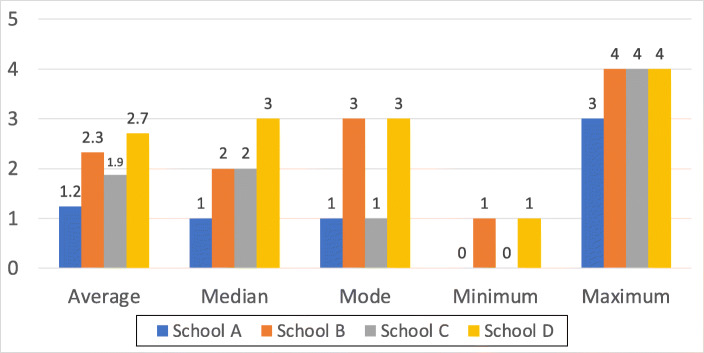
Number of “Best Practice” Principles on Posters (Including ‘Do Not Share’)

The lack of knowledge could have its roots in many factors, ranging from the way their particular school chose to teach them password principles, what devices they used at home, what they saw on TV and what they might have heard from their parents and peers in this respect. The tallies are depicted in Fig. 4.

**Fig. 5:**
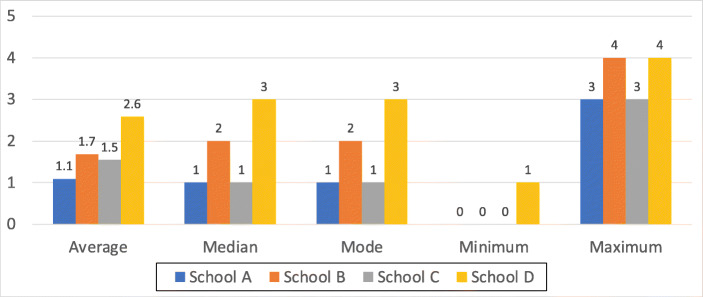
Number of Correct “Best Practice” Principles on Posters (Including ‘Do Not Share’)

Figure 5 then shows which of the principles the children were aware of. The coverage is concerning, reflecting the fact that children were not particularly aware of the latest password “best practice” principles or had assimilated the wrong principles from some environmental source.

### RQ2: Could children create and recall a “silly sentence” password?

The majority of children in all schools were able to create a correct passphrase such as “*Hi im a blue big monster*”. However, the ability to recall the passphrase and replicate it exactly varied (see Table 3). The children from the two schools with the lowest levels of deprivation produced a correct passphrase in far greater numbers than those from the schools with the highest levels of financial deprivation. The differences, when it came to passphrase retention, after only 10 minutes, are even more stark.

**Table 3:** Passphrase Construction and Recall by School

School	Deprivation	% Produced a	% Recalled a	Passphrase
	Level	correct Passphrase^1^	correct Passphrase^2^	strength
A	90-100	54.7	20.8	4.8
B	10-20	92	88.2	5
C	70-80	61.1	27.5	4.69
D	*	85	82.3	4.7

^1^A passphrase made of three words

^2^Percentage of *correct* passphrases

In all schools, the majority of children were able to produce a passphrase, although in School A this was by a narrow margin. Within each school the overall strength of the correct passphrases was good. School A’s average was high on the Bennish calculator, likely due in part to words unwittingly being spelt incorrectly. This was reflected in the recall of passphrase when an alternative spelling was also frequently produced. The most striking differences manifested in the ability to recall and replicate passphrases (Tables 3 and 4).

**Table 4:** Passphrase Examples

Created PassPhrase	Recalled PassPhrase	Coded
Hi 56786575288Hi	Hi605276758852Hi	Not a PassPhrase
the rabit went to the shops to get mony	the rabet went to the shops to get mony	Passphrase recalled with incorrect spelling
My 31st Sudoku Grid	My 31st Sudoku Grid	Passphrase recalled correctly
My silly dog is very very very silly	My silly dog is very very silly	Passphrase not recalled correctly

### RQ3: Did financial deprivation impact children’s knowledge and skills

The school with the most collective knowledge was the School D, while the school with the least knowledge had the highest level of deprivation (School A). School D also had the most posters which contained information other than the advice not to share passwords. One example of guidance from School D was “*Don’t make your hints or password obvious*”, no posters in the other schools made reference to password hints. In this school only one poster did not contain any other information. School A had 30 posters which did not contain any other information (71.4%). Examples of other guidance from School A included “*make it easy to remember*”. School B, the least deprived of the state schools, showed results close to those of School D, with just 3 of the posters containing no other information. It is worth mentioning that School D did not issue passwords to the children before the age of 9. This means that the children are unlikely to have picked up bad habits due to their not being required to use passwords before they are ready to use them (Stewart et al., 2020). However, these children also experienced the lowest levels of financial deprivation, which means that they probably had exposure to Internet-enabled devices at home and learnt good practice from other sources.

The children in School C, which had the second highest level of deprivation, produced 19.5% of posters with no other information apart from the advice not to share passwords.

When developing a passphrase, the state school with the least deprivation (School B) was more likely than the School D to produce a correct phrase, while the two state schools with higher levels of deprivation were the least likely to do so. However, in all schools, the majority of children were able to produce a passphrase after our brief lesson.

Passphrase recall showed that children from least deprived areas were the only ones in which the majority were able to recall their passphrases. The other schools were closely matched on the ability to recall, which was very low.

**In summary,** financial deprivation’s impact is evident. The deprived children did not have the same knowledge levels, and were not able to recall a password they had created a matter of ten minutes before (differing abilities). This stark difference emerged despite the children being the same age, following the same curriculum and being taught the same principles by equally qualified teachers.

## Discussion & reflection

Up-to-date and accurate knowledge about passwords and appropriate password practices is required by all children in the modern world. Our study demonstrated that the chances of a child having these skills and knowledge are lower for those growing up in areas of financial deprivation. This means that these children are already more vulnerable at age 9, and probably unlikely to catch up.

Pupils in the two schools with highest levels of deprivation were not only less likely to be able to share good password practices but were also less likely to be able to recall a strong password.

No one child, nor indeed one class, was able to name all the guidance that children their age should know about passwords. This is not necessarily that unexpected because adults manifest the same deficiencies in this domain ((Prior & Renaud, 2020; Guo 2013). The children’s parents were unlikely to have had this knowledge included in their curriculum when they were at school, and the most deprived schools’ parents were often not in employment, so likely themselves excluded from learning the latest password “best practice” principles (Seabright, 2010). The parents of the children in the other areas are more likely to be able to impart principles to their children.

Hyslop and Keddell (Hyslop & Keddell, 2018) explain that poverty shames and disempowers, and reduces the confidence and competence perceptions of those who are experiencing financial deprivation. A lack of confidence in their own abilities and competence is likely to deter parents from trying to teach their children about a variety of principles, and cyber security might be one of these.

Moreover, Arnup et al. (Arnup et al., 2020) find that deprived children spend significantly more time in front of screens, with passive screen time and excessive screen time being prevalent. Children are unlikely to get up to date information from this kind of activity.

It is clear that more resources are required to improve cybersecurity knowledge and skills and particularly their password “best practice” skills. Otherwise, children from deprived areas will be more vulnerable online despite the best efforts of their teachers and the educational authorities. With children going online in unprecedented numbers as a consequence of the pandemic, we have to bolster the password management skills of all our children.

### Summary

The participating children did not know many password “best practice” principles (Fig. 4), and some of their knowledge was outdated (e.g., complexity requirements) (Fig. 5). Moreover, children in areas of financial deprivation seemed to be least able have knowledge, or be able to retain a passphrase they had created a short time before.

However, it should be highlighted that over half of the children, regardless of financial deprivation, were able to create a passphrase after a lesson. This gives us hope that the situation is redeemable. The children who still struggled might need a bit more time and repetition before they, too, will be able to apply new knowledge. We need an intervention to reach and support teachers by providing resources they can use to teach children the latest password “best practice” principles. In the next section, we propose one intervention for the classroom to be delivered by teachers. It is clear from the results of the study that a targeted extra-curricular intervention is also required for children living in areas of financial deprivation. We therefore propose a second intervention aimed at this group to take place outside of the usual school day.

### Suggested interventions

Nieuwenhuis and Chiang (2021) argue that there does not necessarily have to be a direct link between perceived relative economic disadvantages and student outcomes. It is not a simple matter to neutralise the impact of financial deprivation on children, but it is possible. Rojas-Barahona et al. (2015) find that whereas children from deprived areas had poorer working memory, it was possible to improve this with a well targeted intervention. This finding was confirmed by (McKay et al., 1978; Banerjee, 2016; Ni Shuilleabhain et al., 2020).

Such interventions need to be designed with the target educational environments in mind (Verma and Verma, 1994; McKay et al., 1978). Cybersecurity books have been found to frequently contain outdated or incorrect information (Renaud & Prior, 2021a). While there are several high quality online password education resources available e.g. Google: Play Interland - Be Internet Awesome (), these are either not being used or not having the required impact on password knowledge in Scottish schools.

In terms of improving password-related knowledge and skills in Scotland, we suggest that active learning be explored. Active learning can be defined as “*...an approach to instruction that involves actively engaging students with the course material through discussions, problem solving, case studies, role plays and other methods*” Active Learning: Teaching and learning in higher education (2021). We are also keenly aware of the time pressures faced by teachers, something that has only be exacerbated by the Covid-19 pandemic as teachers attempt to make up for “lost learning time” (Engzell et al., 2021). Thus we believe that the best means of effectively delivering high quality password-related knowledge, without creating additional work for teachers, is for resources to be developed which can easily be used by teachers with minimal preparation time.

However it cannot simply be left to the teachers to narrow the deprivation gap in cybersecurity knowledge. Currently, many local authorities provide free or very inexpensive holiday clubs within deprived areas. These frequently offer children the opportunity to sample different forms of sport, arts and crafts along with a free meal during the school holidays. These are an ideal opportunity for cybersecurity knowledge to be provided in a fun and relaxed environment. By working with local universities who offer volunteering schemes for students, local authorities could harness their skills and expertise, to impart the knowledge to children, and to engage in acttivities to develop their abilities in this domain. The students also gain experience and credit by participating in their volunteering scheme.

### Future work

This study has shown that children from more deprived backgrounds were less likely to be able to successfully remember a passphrase, and also had less knowledge of password “best practice”. This study did not attempt to investigate why this was the case, which should be the focus of a future study. The impacts of deprivation are multifaceted and it is not clear what other factors may explain what we observed.

While the children from schools in the least deprived area were able to provide more password knowledge, no school demonstrated knowing all of the password concepts correctly. It is unrealistic to expect teachers to be able to develop password education resources, so more work is needed in a consultation between experts in password education and educational professionals to develop ready-to-use resources for the classroom.

Zevenbergen and Logan (2008) found gender differences in terms of how computers are being accessed and in skill development, and this was evident by four or five years of age. It would be interesting to test for a gender bias in the password domain too.

Pratt (1978) finds that there is a greater incidence of stress experienced by teachers of children from the more deprived areas. In designing our interventions we ought to co-design with these teachers to ensure that our interventions meet their needs.

### Limitations

**Fig. 6:**
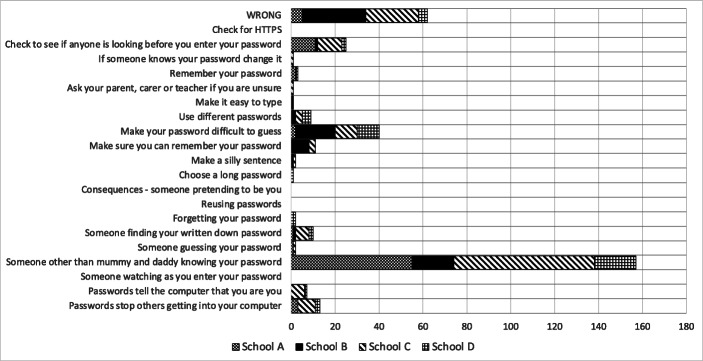
Histogram of Password “best practice” Principles

Much online advice related to password “best practice” is outdated (Renaud and Prior, 2021b; Prior & Renaud, 2020). The relatively poor knowledge demonstrated (Section 4.1) is likely to have been impacted by the fact that much of the population has not yet caught up with the latest guidelines. Yet, this does not explain the differences between the schools (Fig. 6) all working through the same curriculum. This is more likely to be attributable to the differences in financial deprivation experienced by the children.

This study was carried out in Scottish schools in two North Eastern cities. It is possible that teachers in different parts of the UK, or those in other countries, would have a better grasp of “best practice” password management principles. It is also possible that the children in other areas are better informed. However, our focus was on deprivation, and its impact on children. As such, doing all the research in a single domain where all children are taught the same curriculum by teachers registered with the same educational authority removed a number of potentially confounding factors and allowed us to reveal differences that are more likely to be attributable to financial deprivation.

We asked the children to produce poster drawings, which seemed the best way to assess their mental models so that we did not frame responses. Children draw from a young age, so we did not believe this to be an unrealistic expectation. However, our ethical review board required us to tell children not to share their passwords before they commenced. This compromised the study to a certain extent because were were not able to determine whether they already knew this rule before we visited the school. Indeed, almost every child included this advice on their posters. While we are glad that we made the children aware of this good advice, it did mean that we could not gauge how widespread this knowledge had been before our investigation commenced.

This study did not intend to demonstrate statistical significance in the different levels of knowledge in the four schools. A future study looking at the knowledge from a quantitative perspective would deliver interesting insights.

## Conclusion

Current and correct password knowledge is required by all children in the modern world, as is the ability to implement this knowledge in everyday password practices. For an equitable society this knowledge needs to be shared by all children regardless of background. However our study has demonstrated wide variations in knowledge in children living in areas with different levels of financial deprivation.

We find that knowledge of password “best practice” is not widely shared by children by the age of 9. We suggest interventions for schools and also targeted interventions for children living in areas in which we found the lowest level of password knowledge.

This study was undertaken in only four schools, and we do not claim to have enough data to make any claims about the same knowledge and ability levels in other parts of Scotland or indeed the entire UK. Yet, the differences in our study are so marked that a greater study would be well worth pursuing to gain greater insights into the potentially deleterious impact of financial deprivation on the cyber security knowledge of children, and consequently on their greater online vulnerability.

## Footnotes

1 https://www.ons.gov.uk/employmentandlabourmarket/peoplenotinwork/unemployment/timeseries/mgsx/lms

2 https://www.ncsc.gov.uk/cyberaware/home
